# Magnitude, trends and drivers of the coexistence of maternal overweight/obesity and childhood undernutrition in Ethiopia: Evidence from Demographic and Health Surveys (2005–2016)

**DOI:** 10.1111/mcn.13372

**Published:** 2022-05-25

**Authors:** Rebecca Pradeilles, Ana Irache, Tom Norris, Stanley Chitekwe, Arnaud Laillou, Kaleab Baye

**Affiliations:** ^1^ School of Sport, Exercise and Health Sciences (SSEHS) Loughborough University Loughborough UK; ^2^ Warwick Medical School University of Warwick Coventry UK; ^3^ Department of Targeted Intervention, Division of Surgery and Interventional Science University College London London UK; ^4^ Nutrition Section, UNICEF Ethiopia Addis Ababa Ethiopia; ^5^ Center for Food Science and Nutrition Addis Ababa University Addis Ababa Ethiopia; ^6^ Research Center for Inclusive Development in Africa (RIDA) Addis Ababa Ethiopia

**Keywords:** anaemia, children under 5, double burden of malnutrition, drivers, Ethiopia, obesity, overweight, stunting, women of childbearing age

## Abstract

Ethiopia faces a rising problem of overweight and obesity alongside a high prevalence of undernutrition; a double burden of malnutrition (DBM). This study aimed to quantify the magnitude and trends of household‐level DBM—defined as the coexistence of maternal overweight/obesity and child undernutrition (i.e., stunting or anaemia)—in Ethiopia between 2005, 2011 and 2016 and understand the potential drivers influencing DBM and the change in DBM over time. Data come from the Ethiopian Demographic and Health Surveys. National and regional prevalence estimates of the DBM were calculated (*n* = 13,107). Equiplots were produced to display inequalities in the distribution of DBM. Factors associated with DBM were explored using pooled multivariable logistic regression analyses for 2005, 2011 and 2016 (*n* = 9358). These were also included in a logistic regression decomposition analysis to understand their contribution to the change in DBM between 2005 and 2016 (*n* = 5285). The prevalence of household‐level DBM at the national level was low, with a modest increase from 2.4% in 2005% to 3.5% in 2016. This masks important within‐country variability, with substantially higher prevalence in Addis Ababa (22.8%). Factors positively associated with DBM were maternal age (odds ratio [OR] = 1.04 [1.02, 1.06]), urban residence (OR = 3.12 [2.24, 4.36]), wealth (OR = 1.14 [1.06, 1.24]) and the number of children <5 in the household (OR = 1.30 [1.12, 1.49]). Overall, 70.5% of the increase in DBM between 2005 and 2016 was attributed to increased wealth, urban residence and region. Double‐duty actions that address multiple forms of malnutrition are urgently needed in urban settings.

## Key messages

- Despite a low national prevalence, household level DBM is significantly higher in urban areas, particularly in Addis Ababa where nearly a quarter of households are affected.
- Maternal age, urban residence, wealth and the number of children <5 in the household were positively associated with DBM.
- Increased wealth and urban residence were the main drivers associated with increases in DBM between 2005 and 2016.
- Double‐dutyactions addressing multiple forms of malnutrition are required in urban settings.
- Transforming food systems by regulating the promotion and distribution of unhealthy foods and promoting nutrient‐dense foods is critical.

## Graphical abstract

The present study has highlighted that a relatively low prevalence of household‐level double burden of malnutrition (DBM) at the national level can mask serious disparities among subpopulations. The prevalence of household‐level DBM is significantly higher in urban areas and in households with higher wealth, calling for double‐duty actions that address both forms of malnutrition, particularly in urban areas.

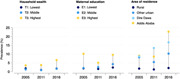

## 1 INTRODUCTION

While progress in reducing undernutrition has been witnessed in most low‐ and middle‐income countries (LMICs), the pace of progress has been too slow, and many countries are still not on track to achieve the nutrition targets set out in the sustainable development goals (FAO et al., 2021). In 2020, globally, 149.2 and 45.4 million children under 5 years of age (U5) were stunted and wasted, respectively, with 462 million adults underweight (WHO, 2021). At the same time, 38.9 million children U5 were overweight or obese globally (WHO, 2021). These two forms of malnutrition (i.e., undernutrition and overweight/obesity) have traditionally been considered as distinct manifestations requiring different interventions (Wells et al., 2020). Interventions addressing undernutrition were prioritised by funders and programme implementers in low‐income countries, leaving overweight and obesity unwatched.

An increasing body of evidence has shown that undernutrition (stunting, wasting and micronutrient deficiencies) and overweight/obesity can coexist, affecting countries, households and individuals (Lerm et al., 2021). This phenomenon has been labelled the double burden of malnutrition (DBM). The two forms of malnutrition are not only an epidemiologic phenomenon but are also biologically interlinked (Mendenhall & Singer, 2019). For example, early life undernutrition has been linked to an increased risk of overweight and obesity in later life. The two forms of malnutrition are also linked by shared drivers like poor diet quality. The recent Lancet Series on the DBM has also shed light on the need to reshape public health and nutrition interventions to address multiple forms of malnutrition simultaneously (Hawkes et al., 2020).

The DBM has been recognised as an important public health problem in sub‐Saharan Africa (SSA) (Reardon et al., 2021). Recent analyses conducted in SSA revealed an increasing burden of mortality attributable to overweight/obesity and dietary risks, in addition to high, but decreasing, mortality attributed to maternal and child undernutrition (Melaku et al., 2019). This calls for interventions addressing both undernutrition and overweight/obesity. A number of observational studies, including regression decomposition studies, have helped identify drivers associated with the change in the prevalence of malnutrition (Baye & Hirvonen, 2020a; Golan et al., 2019; Headey, 2019). These studies were focused on drivers affecting one form of malnutrition only (e.g., stunting) and can now be replicated to identify shared drivers of multiple forms of malnutrition simultaneously. Such decomposition studies can be guided by recent conceptual frameworks that have identified shared drivers of multiple forms of malnutrition (Pradeilles et al., 2019).

Ethiopia presents a great opportunity for this kind of analysis because it still has a high prevalence of undernutrition and a rising burden of overweight and obesity, particularly among women of reproductive age (WRA) in urban areas (Baye & Hirvonen, 2020b). The shift in the burden from undernutrition to overweight/obesity has been reflected in the rise of noncommunicable diseases (NCDs), which alongside the persistently high rates of infectious diseases, is further stretching the health system. Cardiovascular diseases are now the leading cause of age‐standardised mortality in Ethiopia (Baye & Hirvonen, 2020b). Nevertheless, programmes and funding remain primarily focused on undernutrition. Shifting policies and programmes to address both forms of malnutrition requires a good understanding of the magnitude, trends and drivers of DBM. Unfortunately, such evidence is scarce, hindering the much‐needed shift in programmes and policies towards double‐duty actions that address both forms of malnutrition simultaneously.

Therefore, in this paper, we aimed to: (i) assess the prevalence, trends and inequalities in the distribution of household‐level DBM; (ii) explore drivers associated with household‐level DBM; and (iii) understand the potential drivers influencing the change in household‐level DBM over time through a regression‐decomposition analysis.

## 2 METHODS

### 2.1 Study design, sampling and data collection tools

This study uses data from the 2005, 2011 and 2016 rounds of the Ethiopian Demographic and Health Surveys (EDHS), which collected data for both anthropometric and anaemia measures. The EDHS uses a stratified two‐stage cluster sampling method and contains nationally and regionally representative cross‐sectional data for households, including nutritional data among WRA (15–49 years) and children U5. These surveys use standardised procedures (e.g., survey instruments and data collection methods) and are publicly available from https://dhsprogram.com/. Complete descriptions of sampling, data collection methods, questionnaire and data validation procedures are published elsewhere (Croft et al., 2018).

All data are deidentified and ethical approval to conduct the DHS surveys was obtained centrally by the ORC Macro Institutional Review Board and locally. Ethical approval was not sought for this analysis of secondary data.

### 2.2 Data management and analyses

#### 2.2.1 Outcome measurements

The outcome of interest was household‐level DBM, defined as the coexistence of maternal overweight/obesity and child undernutrition (i.e., stunting or anaemia). Anthropometric measurements (weight and height/length) in mothers and their children were taken using standard procedures by trained fieldworkers (ICF International, 2012). Weight was measured in light clothing to the nearest 0.1 kg using a lightweight SECA digital scale designed and manufactured under the guidance of the United Nations Children's Fund (UNICEF). The weight of younger children was obtained through an automatic mother–child adjustment that eliminated the mother's weight while she was standing on the scale with her child. Maternal and infant height/length were measured to the nearest 0.1 cm using a Shorr Productions measuring board. Recumbent length was measured in children younger than 24 months or in special circumstances (i.e., when the child's age was not known and the child was less than 85 cm tall), while standing height was measured in older children. We excluded anthropometric data from mothers who were pregnant or who had given birth in the 2 months preceding data collection (Croft et al., 2018). Overweight/obesity was defined as a body mass index (BMI) ≥ 25.0 kg/m^2^ for adult women (20–49 years old) and as a BMI‐for‐age *z*‐score > 1 SD above the median of the reference for adolescent girls (15–19 years old) (WHO, 2007, 2020). Stunting among children U5 was defined as length/height‐for‐age *z*‐score < −2 SD below the median of the reference (WHO, 2006). Haemoglobin levels were measured among children aged 6–59 months using HemoCue® administered either at the end of a finger or for infants aged less than 12 months, at the heel. Anaemia was defined as a haemoglobin level (adjusted for altitude) <11.0 g/dl (Pullum et al., 2017).

For this study, we created three binary variables to quantify three possible types of household‐level DBM: (1) maternal overweight/obesity and U5 stunting (type 1); (2) maternal overweight/obesity and U5 anaemia (type 2); and (3) maternal overweight/obesity and U5 anaemia and/or stunting (type 3). Forms of undernutrition did not include wasting as the household level prevalence of maternal overweight/obesity and child wasting was close to zero in 2005 (0.2%), 2011 (0.4%) and 2016 (0.2%). The prevalence of each form of malnutrition at the population level (i.e., maternal overweight, U5 stunting, U5 anaemia, U5 wasting) and of the different possible combinations at the household level are presented in Supporting Information: Tables 1 and 2, respectively.

We calculated household‐level DBM using data available for all mothers and children U5 living within the same household. When one mother with overweight/obesity had two or more children with a form of undernutrition, we counted this as one household presenting with DBM (i.e., one case of DBM). The same logic was applied for instances where two or more mothers with overweight/obesity were living in the same household, both with undernourished children. The main analyses are based on any form of household‐level DBM (i.e., maternal overweight/obesity and U5 anaemia and/or stunting), with types 1 and 2 presented as supplementary analyses.

#### 2.2.2 Exposure measurements

Exposure variables included shared drivers of malnutrition (i.e., those that influence simultaneously overweight/obesity and undernutrition (stunting or anaemia)). We first started by listing all potential shared drivers of multiple forms of malnutrition at any level, using an existing framework for DBM (Pradeilles et al., 2019). We then checked which variables were available within the DHS. Finally, we decided to only retain factors that could influence maternal and child nutritional status simultaneously, that is, maternal‐ or household‐level variables described as follows:

– *Regional level variables*: The two main variables included were the area of residence (urban vs. rural) and regions (Tigray, Afar, Amhara, Oromia, Somali, Benishangul, Southern Nations, Nationalities, and Peoples' Region, Gambela, Harari, Addis Ababa, Dire Dawa). The area of residence was defined according to the country‐specific definition and initially categorised as urban or rural. We hypothesised that the prevalence of household‐level DBM might differ in the capital or main cities in Ethiopia; thus, we further categorised the area of residence for part of the analysis into: Addis Ababa (city), Dire Dawa (city) and other urban areas and rural areas.
– *Household‐level variables*: A household wealth index was created for each time point (2005, 2011 and 2016) using factor analysis applied to proxy indicators of the household environment (ownership of consumer durables; house ownership; land ownership; main source of energy for cooking; livestock ownership; electricity; source of drinking water and type of toilet facilities; number of rooms used for sleeping; type of materials used for floor, roof and walls). Given the differences in living conditions and in ownership of assets between urban and rural areas, we decided to run the factor analysis separately for the two settings. Variables with low variability (i.e., either less than 5% or more than 95% of ownership) were not included. A common household wealth index was then created and tertiles were derived (T1, T2 and T3), with the first tertile representing the relatively poorest one. Internal validity was assessed by tabulating ownership of durable assets and housing characteristics by SES tertile. The time‐specific wealth indices were used to display inequalities in the distribution of household‐level DBM. For the analysis of factors associated with household‐level DBM, we created a pooled household wealth index (a continuous measure that we rescaled to 0–10) using data from 2005, 2011 and 2016 following the same methodology than the one described above. For the decomposition analysis, the same was performed but using data from 2005 to 2016 only. Besides wealth, we also included variables such as the number of children aged less than 5 years (including the reference child), number of WRA per household, sex of household head, and age of household head.
– *Mother‐specific variables*: Maternal education was based on mothers’ self‐report of completed educational level and categorised into three levels (E1: no education; E2: primary education; E3: secondary education or higher). In the decomposition analysis, age was used as a continuous measure (i.e., number of years of schooling). Literacy (cannot read at all, able to read only parts of a sentence and able to read a whole sentence), occupational status (not working, nonmanual and manual), maternal age and women's empowerment were also used in the analysis. Women's empowerment was quantified using the recent survey‐based women's empowerment index (SWPER) developed and validated for use in Africa (Ewerling et al., 2017). SWPER scores for (i) attitude towards violence, (ii) social independence (autonomy) and (iii) decision‐making domains of empowerment were calculated. The indices are generated based on the DHS empowerment module that assesses women's involvement in household decisions, employment and type of earnings, control of resources, opinion on partner violence, and ownership of house land. Details related to the construction of the SWPER are presented in Supporting Information: Table 3. In this analysis, we only included the scores for social independence (autonomy) and decision‐making, based on earlier findings (Baye et al., 2021).
– *Father‐specific variables*: Paternal education (i.e., number of years of schooling) and occupational status (not working, nonmanual and manual) were included in the analysis.

### 2.3 Statistical analysis

#### 2.3.1 Prevalence data, trends and inequalities in the distribution of household‐level DBM

National prevalence estimates and 95% confidence intervals (CIs) of the three types of household‐level DBM for the years 2005, 2011 and 2016 were first calculated and then by region. Sampling weights were applied when estimating the prevalence of outcomes to account for the sampling design used in the EDHS. Equiplots were then produced to display inequalities in the distribution of household‐level DBM by household wealth, maternal education and area of residence. We measured inequality gaps for each survey round, defined as the absolute difference between: (1) the DBM prevalence in the lowest wealth tertile versus the highest wealth tertile, (2) the DBM prevalence in the lowest education level versus the highest education level, and (3) the DBM prevalence in rural areas versus urban areas. Accordingly, a positive difference indicates a higher prevalence of household‐level DBM in the lowest wealth tertile, lowest educational level and in rural areas.

#### 2.3.2 Factors associated with household‐level DBM

Associations between regional‐, household‐, mother‐ and father‐level variables (i.e., the main exposure variables identified as shared drivers of DBM) and household‐level DBM were examined using univariable and multivariable logistic regressions. Each exposure variable in the multivariable models was mutually adjusted for all other variables. Separate regressions were also performed to assess the relationships between shared drivers and individual forms of malnutrition (U5 anaemia, U5 stunting and maternal overweight/obesity). The analytical sample was limited to households with complete data on maternal overweight/obesity and child undernutrition (i.e., anaemia or stunting).

#### 2.3.3 Decomposition analysis

For the decomposition analysis, we restricted the sample to only include the earliest and latest time points (2005 and 2016). As some households included multiple mothers, we randomly selected one mother per household. Following this, we recalculated DBM type 3 (i.e., maternal overweight/obesity and U5 stunting or anaemia) on the restricted sample.

Before running the decomposition analysis, we measured the change in the means of explanatory factors between 2005 and 2016. Variables that did not change over time were not included in the decomposition analysis.

To understand the contribution of selected shared drivers to the change in household‐level DBM (defined as a mother overweight/obese and a child either stunted or anaemic) between 2005 and 2016, we utilised a nonlinear multivariate regression decomposition method based on logistic regression models adapted from the Oaxaca–Blinder technique (Blinder, 1973; Oaxaca, 1973). These were based on those with the complete outcome and covariate data.

This method allowed us to decompose the observed differences in the prevalence of DBM between 2005 and 2016 into two components. Equation (1) shows the decomposition of the observed difference in DBM prevalence between 2016 (comparison group) and 2005 (reference group):

(1) $$\begin{array}{l} {\bar{Y}}_{2016}-{\bar{Y}}_{2005}=G\left(X\prime {\hat{\beta }}_{2016}|D_{2016}=1\right)-G\left(X\prime {\hat{\beta }}_{2005}|D_{2005}=1\right)\\ \;=G\underset{\text{Explained}\;\text{component}}{\underbrace{\left(X\prime {\hat{\beta }}_{2005}|D_{2016}=1\right)-G\left(X\prime {\hat{\beta }}_{2005}|D_{2005}=1\right)}}\\ \;+G\underset{\text{Unexplained}\;\text{component}}{\underbrace{\left(X\prime {\hat{\beta }}_{2016}|D_{2016}=1\right)-G\left(X\prime {\hat{\beta }}_{2005}|D_{2016}=1\right)},} \end{array}$$

where $D_{2016}$ and $D_{2005}$ represent indicators for the years 2016 and 2005, respectively. *X* is the vector of shared household drivers described previously. *G* (*X*′*β*) describes the mean function of the outcome *Y*, here a dichotomous outcome (DBM) and thus is a logit function. The vectors *β* are estimated separately for 2016 and 2005. The first component of Equation (1) is explained by compositional differences between 2016 and 2005 (i.e., differences in characteristics), while the second is the unexplained component, which derives from differences in the effects of characteristics (i.e., differences in the coefficients). As such the vector of shared household drivers is only those which demonstrated a change between 2005 and 2016, be it a change in the mean or median for continuous variables or a change in prevalence for categorical variables. These were identified using *t* tests and tests on equality of proportions for continuous and categorical variables, respectively.

The above decomposition approach was developed and has typically been applied to linear regression models; however, extensions of the Oaxaca–Blinder approach for use with nonlinear models have been proposed (Bauer & Sinning, 2008; Jann, 2006, 2008; Powers et al., 2011; Yun, 2004). We applied the nonlinear multivariate decomposition method proposed by Powers et al. (2011) using the ‘mvdcmp’ command in Stata. One of the advantages of this method is that the results are insensitive to the order to which the predictors are inserted into the decomposition (path dependency problem). They also overcome the omitted group (Fortin et al., 2011) or identification (Yun, 2005) problem associated with the choice of reference group when categorical variables are included among the predictors. Another advantage of the command is that it produces a detailed decomposition that returns the relative contribution of each predictor along with robust standard errors for the explained and unexplained components.

Separate decomposition analyses for U5 stunting and maternal overweight/obesity were also performed as supplementary analyses. This could not be conducted for U5 anaemia as the prevalence of anaemia did not substantially change between 2005 and 2016. The analytical sample was limited to households with complete data on maternal overweight/obesity and U5 stunting or anaemia.

Robust standard errors were used to account for clustering for both the logistic and decomposition regression analyses. All analyses were conducted on Stata version V.16.0 (Statacorp).

## 3 RESULTS

### 3.1 Characteristics of surveys and households

The analysis included a total of 13,107 households with data on maternal overweight/obesity and child stunting or anaemia across the three time points (2005, 2011 and 2016). There were 12,579 households with available data on maternal overweight/obesity and U5 stunting, and 11,169 households with data on maternal overweight/obesity and U5 anaemia. The participants’ flow diagram is presented in Supporting Information: Figure 1. Sociodemographic characteristics of households with available data on both maternal overweight/obesity and U5 anaemia or stunting are presented in Table 1.

**Table 1:** Sample characteristics (unit household level).

	2005 EDHS, *n* (%)	2011 EDHS, *n* (%)	2016 EDHS, *n* (%)
**Region**			
Tigray	244 (10.3)	655 (11.8)	618 (11.9)
Afar	145 (6.1)	477 (8.6)	418 (8.0)
Amhara	353 (14.8)	744 (13.4)	613 (11.8)
Oromia	424 (17.8)	798 (14.5)	749 (14.4)
Somali	138 (5.8)	346 (6.3)	518 (9.9)
Benishangul	176 (7.4)	473 (8.6)	411 (7.9)
SNNPR	415 (17.5)	765 (13.9)	679 (13.0)
Gambela	133 (5.6)	409 (7.4)	385 (7.4)
Harari	117 (4.9)	292 (5.3)	263 (5.1)
Addis Ababa	120 (5.0)	251 (4.5)	288 (5.5)
Dire Dawa	113 (4.8)	312 (5.7)	265 (5.1)
**Wealth tertiles**			
Poorest (T1)	1034 (43.5)	2563 (46.4)	2518 (48.3)
Middle (T2)	404 (17.0)	895 (16.2)	779 (15.0)
Richest (T3)	940 (39.5)	2064 (37.4)	1910 (36.7)
**Maternal education**			
No education (E1)	1758 (73.9)	3706 (67.1)	3136 (60.2)
Primary (E2)	396 (16.7)	1477 (26.8)	1431 (27.5)
Secondary+ (E3)	224 (9.4)	339 (6.1)	640 (12.3)
**Area of residence**			
Addis Ababa (city)	111 (4.7)	251 (4.6)	288 (5.5)
Dire Dawa (city)	62 (2.6)	140 (2.5)	139 (2.7)
Other urban areas	230 (9.7)	670 (12.1)	693 (13.3)
Rural areas	1975 (83.0)	4461 (80.8)	4087 (78.5)
**Total # of households**	**2378**	**5522**	**5207**

Abbreviations: EDHS, Ethiopian Demographic and Health Survey; SNNPR, Southern Nations, Nationalities, and Peoples' Region.

### 3.2 The magnitude of the DBM at the population level

The prevalence of overweight/obesity among WRA was 8.1% in the most recent data set (2016), increasing from 4.7% in 2005. Stunting in children U5 declined over time (from 49.7% in 2005% to 38.0% in 2016), while anaemia remained relatively stable from 52.2% in 2005% to 57.0% in 2016 (Supporting Information: Table 1).

### 3.3 The magnitude and distribution of household‐level DBM

#### 3.3.1 Household‐level DBM at the national level

The prevalence of any form of household‐level DBM at the national level was low, with a modest increase from 2.4% in 2005% to 3.5% in 2016 (Supporting Information: Table 2). The most recent survey showed that the prevalence of maternal overweight/obesity and U5 anaemia was higher than that of maternal overweight/obesity and U5 stunting (3.1% vs. 1.5%). However, estimates varied between regions and by sociodemographic characteristics.

#### 3.3.2 Regional differences in household‐level DBM

**Figure 1:**
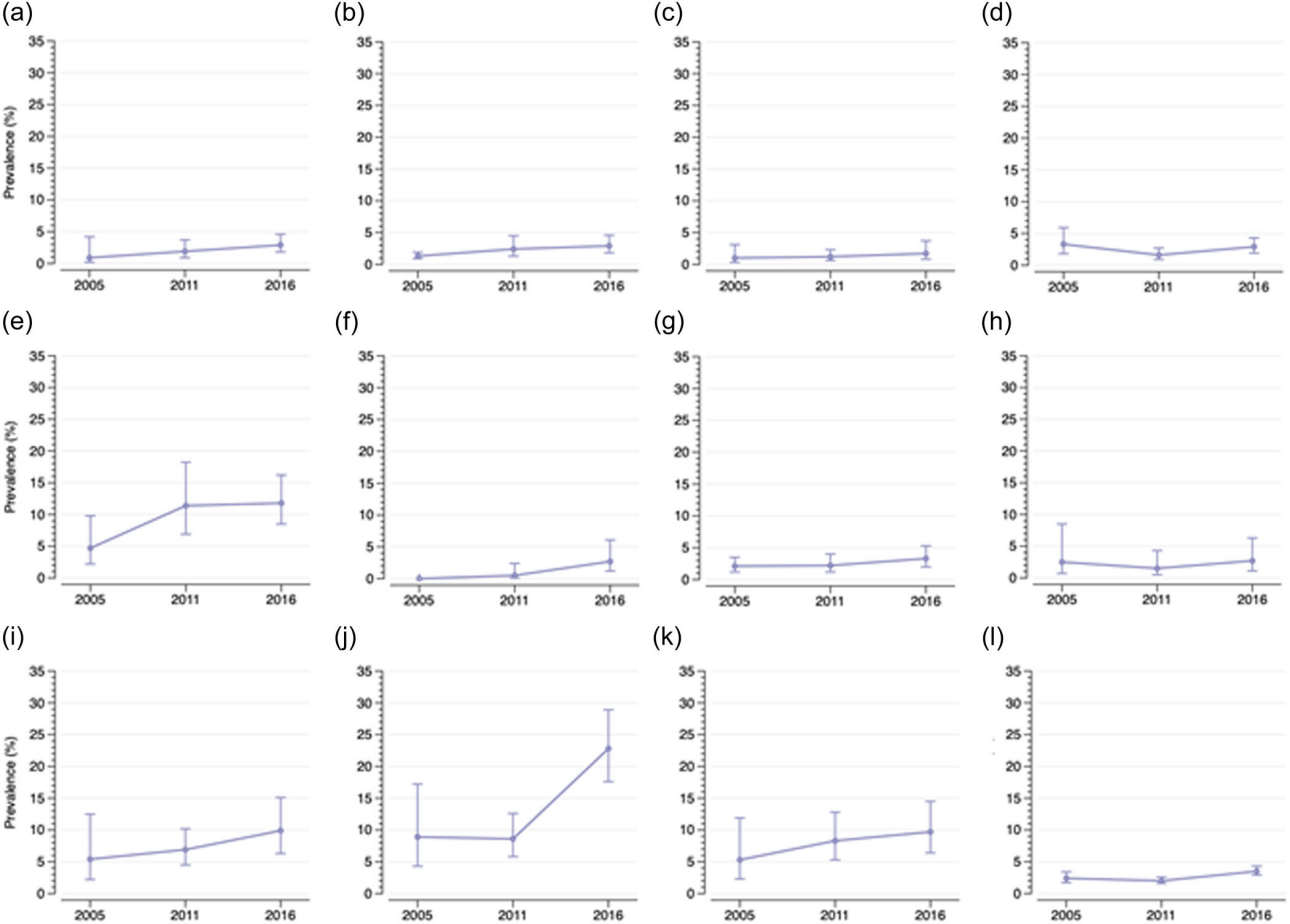
Trends (prevalence and 95% confidence intervals) in regional and national estimates of any household‐level double burden of malnutrition. (a) Tigray, (b) Afar, (c) Amhara, (d) Oromia, (e) Somali, (f) Benishangul, (g) Southern Nations, Nationalities, and Peoples' Region, (h) Gambela, (i) Harari, (j) Addis Ababa, (k) Dire Dawa and (l) National.

Figure 1 shows how the magnitude of household‐level DBM differs across the 11 regions, and how this has changed over time. Using the most recent sweep, the prevalence ranged from 1.7% in Amhara to 22.8% in Addis Ababa for any form of DBM; 0.7% in Amhara to 5.4% in Dire Dawa for maternal overweight/obesity and U5 stunting; and 1.5% in Amhara to 24.9% in Addis Ababa for maternal overweight/obesity and U5 anaemia (Supporting Information: Table 2). There were relatively small differences (<3%‐points) in the regional prevalence estimates of any form of DBM between 2005 and 2016, although a few exceptions were present. For example, in Addis Ababa, prevalence more than tripled from 8.9% in 2005 to 22.8% in 2016, with the highest increase over the period 2011–2016. The annualised change values suggest that the increase in the prevalence of any household‐level DBM is driven primarily by increases in maternal overweight/obesity and U5 anaemia, whereas maternal overweight/obesity and U5 stunting is increasing at a slower pace or decreasing in some regions (Supporting Information: Table 2).

#### 3.3.3 Sociodemographic differences in household‐level DBM

**Figure 2:**
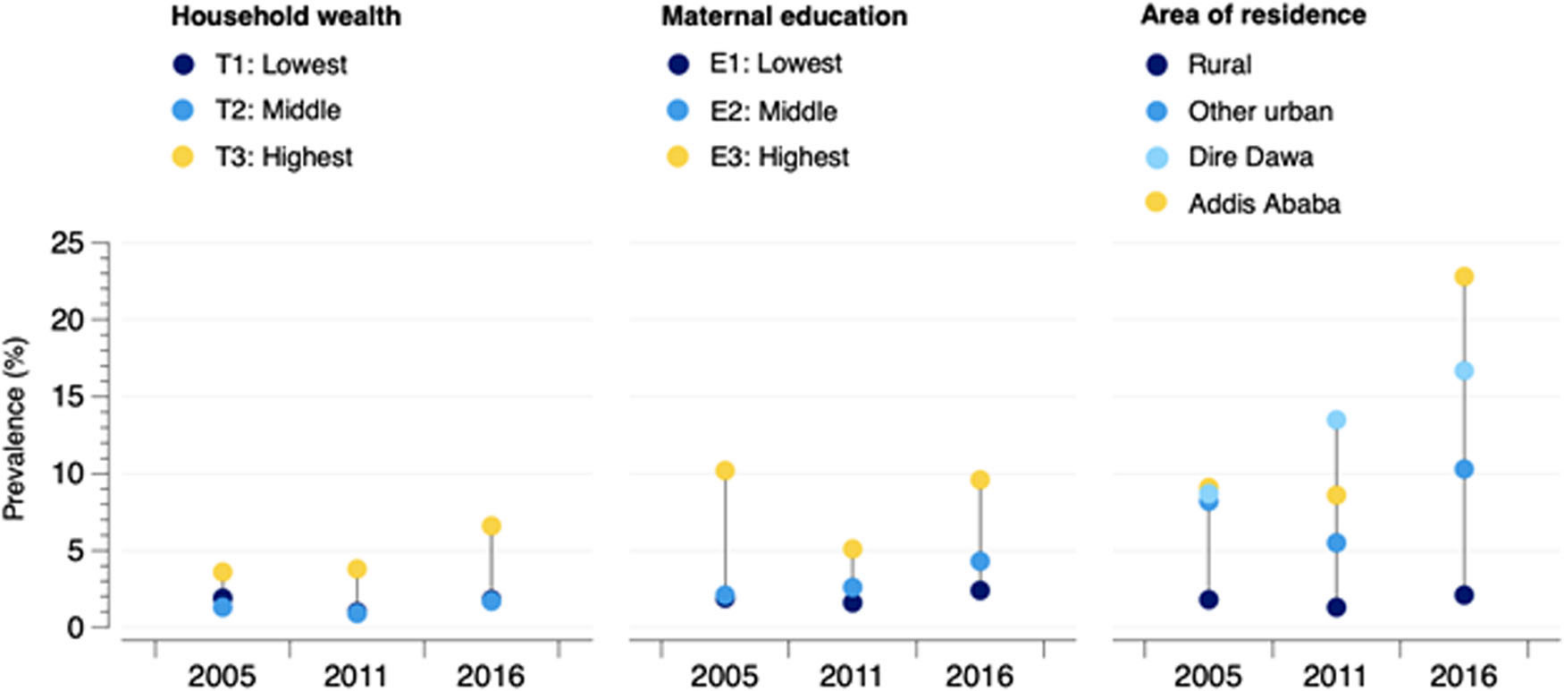
Inequalities in the distribution of the household‐level double burden of malnutrition by household wealth, maternal education level and area of residence.

Socioeconomic disparities in the prevalence of household‐level DBM are displayed in Figure 2. The prevalence of any form of household‐level DBM was highest in the highest wealth tertile (T3) and highest maternal education level (E3) across each of the survey sweeps. Between 2005 and 2016, the inequality gap between the highest and the lowest household wealth tertiles increased (2005 gap: −1.7%‐points vs. 2016 gap: −4.8%‐points), whereas the inequality gap between the highest and lowest maternal education level decreased (2005 gap: −8.3%‐points; 2016 gap: −7.2%‐points) (Supporting Information: Tables 4 and 5). By area of residence, the highest prevalence of any form of DBM was observed in urban areas, with the greatest burden concentrated in the main cities of Dire Dawa and Addis Ababa. The difference in the prevalence between urban and rural areas increased over time (gap: −6.6%‐points in 2005 vs. −10.8%‐points in 2016). Inequalities in the distribution of maternal overweight/obesity and U5 anaemia and maternal overweight/obesity and U5 stunting are presented in Supporting Information: Tables 4–6.

### 3.4 Factors associated with household‐level DBM

In the univariable analysis, factors positively associated with household‐level DBM were maternal age, age of household head, urban residence, maternal literacy, maternal and paternal education, nonmanual occupation for both mothers and fathers, wealth, female‐headed households, number of WRA in the household and women's autonomy/social independence and decision‐making. Maternal/paternal manual occupations were negatively associated with DBM (Table 2). After adjusting for covariates, maternal age (odds ratio [OR] = 1.04 [1.02, 1.06]), urban residence (OR = 3.12 [2.24, 4.36]) and wealth (OR = 1.14 [1.06, 1.24]) remained positively associated with DBM. The number of children <5 in the household (OR = 1.30 [1.12, 1.49]) was positively associated with DBM in the multivariable model only. Univariable and multivariable associations between identified factors and individual forms of malnutrition (U5 stunting, U5 anaemia and maternal overweight/obesity) are presented in Supporting Information: Tables 7 and 8.

**Table 2:** Factors associated with household‐level double burden of malnutrition: pooled univariable and multivariable regression models of EDHS 2005, 2011 and 2016.

Factors	DBM unadjusted estimates (*n* = 11,080)			DBM adjusted estimates (*n* = 9358)		
	OR	95% CI	*p* Value	aOR	95% CI	*p* Value
Maternal age (years)	1.03	1.02, 1.04	<0.001	1.04	1.02, 1.06	<0.001
Residence: Urban	6.96	5.81, 8.34	<0.001	3.12	2.24, 4.36	<0.001
Maternal literacy						
Cannot read at all	Ref.	_	_	Ref.	_	_
Able to read only parts of sentence	1.80	1.34, 2.42	<0.001	1.39	0.95, 2.05	0.091
Able to read whole sentence	3.70	3.05, 4.49	<0.001	1.28	0.80, 2.04	0.30
Maternal Occupation						
Not working	Ref.	_	_	Ref.	_	_
Nonmanual	1.66	1.35, 2.03	<0.001	0.95	0.74, 1.23	0.727
Manual	0.38	0.29, 0.50	<0.001	0.76	0.54, 1.06	0.112
Paternal occupation						
Not working	Ref.	_	_	Ref.	_	_
Nonmanual	1.46	1.02, 2.08	0.037	0.87	0.57, 1.34	0.544
Manual	0.36	0.25, 0.51	<0.001	0.67	0.44, 1.00	0.053
Wealth score (0–10)	1.46	1.41, 1.51	<0.001	1.14	1.06, 1.24	<0.001
Number of children <5 in household	1.09	0.97, 1.22	0.164	1.30	1.12, 1.49	<0.001
Number of WRA in household	1.52	1.37, 1.69	<0.001	1.12	0.96, 1.29	0.134
Sex of household head: Female	1.49	1.22, 1.82	<0.001	1.13	0.87, 1.47	0.353
Age of household head (years)	1.01	1.00, 1.02	<0.001	1.01	0.99, 1.01	0.074
Maternal education (years)	1.15	1.13, 1.17	<0.001	1.01	0.96, 1.06	0.516
Paternal education (years)	1.12	1.11, 1.14	<0.001	1.01	0.98, 1.04	0.436
SWPER: Autonomy/Social independence	1.79	1.63, 1.96	<0.001	0.91	0.78, 1.06	0.230
SWPER: Decision making	1.63	1.46, 1.81	<0.001	1.14	0.99, 1.29	0.055

*Note*: Each variable in the multivariable models was mutually adjusted for all other variables including regions.

Abbreviations: aOR, adjusted odds ratios; CI, confidence interval; DBM, double burden of malnutrition; OR, odds ratios; Ref., reference; SWPER, survey‐based women's empowerment index; WRA, women of reproductive age.

### 3.5 Contribution of the shared drivers to the change in household‐level DBM between 2005 and 2016

Table 3 shows the change in the means of explanatory factors between 2005 and 2016 (*n* = 7580). We note significant increases in sociodemographic characteristics over time. Maternal education increased the most (77.9%) from 1.45 to 2.59 years. Female literacy levels (i.e., able to read only parts of a sentence) increased by 57.1% (from 7.0% to 11.0%). Paternal education also increased from 2.62 years to 4.00 years (52.7% increase). The proportion of mothers and fathers in the occupation increased over time, with the biggest increase seen in nonmanual activities (about 40.0% increase in both sexes). The proportion of female‐headed households increased by 29.4%. There was also a marked change in dimensions related to women's empowerment, particularly autonomy (31.9%). The increase in decision‐making capabilities was relatively small (7.0%) suggesting gender inequity persists.

**Table 3:** Change in the means of key explanatory variables between 2005 and 2016.

	2005 (*n* = 2373)	2016 (*n* = 5207)	Change (2005–2016)		
	Mean (SE)	Mean (SE)	Mean (SE)	% Change (2005–2016)	*p* (diff)
**Continuous variables**					
Wealth score (0–10)	4.26 (0.03)	5.04 (0.02)	0.78 (0.04)	18.3	<0.001
Number of children under 5	1.64 (0.01)	1.60 (0.01)	−0.04 (0.02)	2.4	0.011
Number of WRA in household	1.28 (0.01)	1.25 (0.01)	−0.04 (0.01)	3.1	0.007
Age of household head	38.75 (0.24)	38.44 (0.17)	−0.31 (0.29)	0.8	0.299
Maternal age (years)	29.66 (0.15)	29.72 (0.09)	0.05 (0.17)	0.2	0.766
Maternal education (years)	1.45 (0.06)	2.59 (0.06)	1.13 (0.09)	77.9	<0.001
Paternal education (years)	2.62 (0.08)	4.00 (0.07)	1.38 (0.12)	52.7	<0.001
SWPER: social independence	−0.47 (0.02)	−0.32 (0.01)	0.15 (0.02)	31.9	<0.001
SWPER: decision making	0.04 (0.02)	0.32 (0.01)	0.28 (0.02)	7.0	<0.001
**Categorical variables**					
Residence: Urban	0.17 (0.01)	0.21 (0.01)	0.04 (0.01)	23.5	<0.001
Region					
Tigray	0.10 (0.01)	0.12 (0.00)	0.02 (0.01)	20.0	0.044
Afar	0.06 (0.00)	0.08 (0.00)	0.02 (0.00)	33.3	0.002
Amhara	0.15 (0.01)	0.12 (0.00)	−0.03 (0.01)	20.0	<0.001
Oromia	0.18 (0.01)	0.14 (0.00)	−0.04 (0.01)	22.2	<0.001
Somali	0.06 (0.00)	0.10 (0.00)	0.04 (0.01)	66.7	<0.001
Benishangul	0.07 (0.00)	0.08 (0.00)	0.01 (0.01)	14.3	0.433
SNNPR	0.17 (0.01)	0.13 (0.00)	−0.04 (0.01)	23.5	<0.001
Gambela	0.06 (0.00)	0.07 (0.00)	0.01 (0.00)	16.7	0.004
Harari	0.05 (0.00)	0.05 (0.00)	0.00 (0.00)	0.0	0.824
Addis Ababa	0.05 (0.00)	0.05 (0.00)	0.00 (0.00)	0.0	0.396
Dire Dawa	0.05 (0.00)	0.05 (0.00)	0.00 (0.00)	0.0	0.544
Sex of household head Female	0.17 (0.01)	0.22 (0.01)	0.05 (0.01)	29.4	<0.001
Maternal literacy					
Cannot read at all	0.78 (0.01)	0.71 (0.01)	−0.07 (0.01)	8.9	<0.001
Able to read only parts of sentence	0.07 (0.00)	0.11 (0.00)	0.04 (0.01)	57.1	<0.001
Able to read whole sentence	0.15 (0.01)	0.18 (0.00)	0.03 (0.01)	20.0	<0.001
Maternal occupation					
Not working	0.66 (0.01)	0.56 (0.01)	−0.10 (0.01)	15.1	<0.001
Nonmanual	0.12 (0.01)	0.17 (0.00)	0.05 (0.01)	41.7	<0.001
Manual	0.21 (0.01)	0.27 (0.01)	0.06 (0.01)	28.6	<0.001
Paternal occupation					
Not working	0.01 (0.00)	0.10 (0.00)	0.09 (0.00)	9.0	<0.001
Non manual	0.15 (0.01)	0.21 (0.01)	0.06 (0.01)	40.0	<0.001
Manual	0.84 (0.01)	0.69 (0.01)	0.15 (0.01)	17.9	<0.001

*Note*: *t* test was performed for continuous variables and *χ* ^2^ for categorical/binary variables.

Abbreviations: SNNPR, Southern Nations, Nationalities, and Peoples' Region; WRA, women of reproductive age.

**Figure 3:**
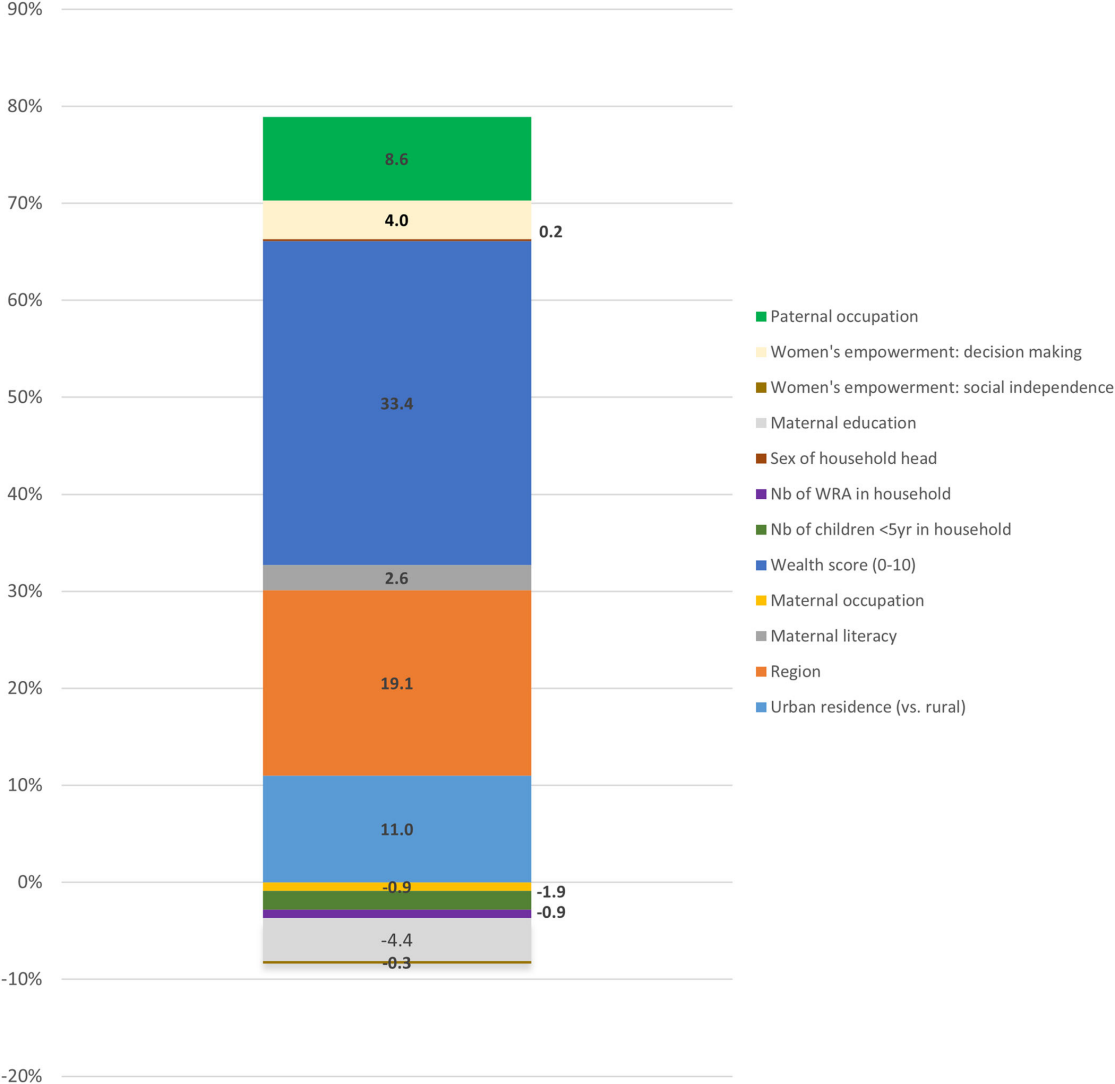
Estimated contributions (%) of selected factors to changes in the likelihood of household‐level double burden of malnutrition between 2005 and 2016.

Figure 3 shows the estimated contributions of selected factors to changes in the likelihood of household‐level DBM (*n* = 5285). Overall, 70.5% of the change in the prevalence of DBM was attributed to changes in the selected characteristics between 2005 and 2016, with the remaining 29.5% unexplained, that is, deriving from differences in the effects of characteristics.

The three factors which contributed the most to the change in the prevalence of DBM and for which the evidence was strongest were wealth, region and urban residence. Increased wealth contributed 33.4% of the increase in the prevalence of DBM (*p* < 0.001). Changes in the prevalence of residing in certain regions contributed to 19.1% of the change in DBM (*p* < 0.001). The increase in the prevalence of urban residence was responsible for 11.0% of the change in the prevalence of DBM (*p* < 0.001). The strength of the evidence for the remaining factors contributing to the increase in DBM was weaker. Types of paternal occupation, women's decision‐making capabilities, maternal literacy levels and sex of household head were responsible for 8.6% (*p* = 0.16), 4.0% (*p* = 0.30), 2.6% (*p* = 0.44) and 0.2% (*p* = 0.91) of the change in DBM, respectively.

Conversely, changes in the number of children U5 and WRA in the household contributed to 1.9% (*p* = 0.02) and 0.9% (*p* = 0.03) of the reduction in DBM, respectively. The strength of the evidence for the remaining factors contributing to the decrease in DBM was weaker. Maternal education (i.e., number of years), types of maternal occupation, and women's autonomy/social independence contributed to a reduction of 4.4% (*p* = 0.36), 0.9% (*p* = 0.56) and 0.3% (*p* = 0.89), respectively. Decomposition analyses for individual forms of malnutrition (U5 stunting, maternal overweight/obesity) are presented in Supporting Information: Table 9.

## 4 DISCUSSION

This study provides an in‐depth and comprehensive assessment of the magnitude, trends, distribution, and drivers of household‐level DBM (defined as maternal overweight/obesity and U5 stunting or anaemia) in Ethiopia using three rounds of representative national surveys. It fills an important gap in the current body of evidence in Ethiopia, which primarily focuses on analysing DHS data to study population‐ (Ahmed et al., 2020) or individual‐level DBM (Farah et al., 2021).

The nutrition landscape of countries like Ethiopia has changed dramatically. Ethiopia has shown a rapid reduction in the prevalence of child stunting (Tasic et al., 2020), but also witnessed rapid increases in overweight and obesity among WRA, particularly in urban settings (Ahmed et al., 2020). In 2016, about one in five (21%) WRA residing in urban areas were overweight or obese, compared with only 4% in rural Ethiopia (Baye & Hirvonen, 2020b). At the same time, anaemia among WRA remained stagnant at 23.9%, with important geographical disparities (Development Initiatives, 2021; Kibret et al., 2019), while child anaemia experienced an important increase between 2011 and 2016 (Amaha, 2020). The prevalence of household‐level DBM at the national level was low, with a modest increase from 2.4% to 3.5% over the period 2005–2016. In 2016, the combination of maternal overweight/obesity and U5 anaemia was more prevalent (3.1%) than the combination of maternal overweight/obesity and U5 stunting (1.5%). This shows that, in our study, household‐level DBM (i.e., maternal overweight/obesity and any form of child undernutrition) was primarily driven by the combination of maternal overweight/obesity and child anaemia. The national prevalence of household‐level DBM, although relatively low, masks important within‐country variability. In 2016, the highest prevalence of household‐level DBM was observed in Addis Ababa (22.8%), with Dire Dawa, Harari, and Somali exhibiting a prevalence of approximately 10%. A recent analysis of anthropometric data across 126 LMICs (1999–2017) revealed that the prevalence of household‐level DBM ranged from 3% to 35%, with the most prevalent form being maternal overweight/obesity and child stunting (Popkin et al., 2020). Similarly, Irache et al. (2022), using anthropometric and biological data, found the form maternal overweight/obesity and childhood anaemia to be the most prevalent form of household‐level DBM, ranging from 3.1% to 42.2%. Countries in LMICs classed in the lowest‐income quartile like Ethiopia are less likely to experience household‐level DBM in comparison to countries in the middle‐income quartiles and more likely to experience population‐level DBM (Popkin et al., 2020). Ethiopia with its fast‐growing economy is aiming to reach lower‐middle‐income status by 2025. Hence, the coexistence of multiple forms of malnutrition at the household level is likely to rise in the coming years.

Wealth and urban residence were the main drivers associated with the household level DBM and increases in DBM over time. In contrast, the increase in maternal education a reflection of the bold government commitments and interventions over the last decades was associated with a decrease in household‐level DBM, although the strength of evidence in support of this association was weak. Rising incomes (wealth score) and urbanisation have been the main drivers of the 'nutrition transition' globally, but also in Ethiopia. Urbanisation is associated with significant lifestyle changes including reduced physical activity, increased proportion of women working and increased demand for more convenient foods, shifting diets from unrefined/minimally processed to highly processed/packaged foods that are often energy‐dense and nutrient‐poor. These urbanisation‐related changes will be further amplified by the projected increase in urbanisation over the coming decades, particularly if the obesogenic behaviours observed in younger generations (e.g., adolescents) remain unchecked (Trübswasser et al., 2021). Studies have shown that income and urbanisation interact in shaping food choices. This, alongside changing food environments where retail stores selling packaged foods become dominant, can accelerate the nutrition transition by shaping food choices, first in urban areas, but eventually in more remote and rural areas (NCD Risk Factor Collaboration NCD‐RisC, 2019; Popkin et al., 2020; Reardon et al., 2021; Trübswasser et al., 2021). Our findings of an increasing prevalence of household‐level DBM in urban settings are in line with recent findings from a multicountry analysis of SSA examining the relationship between urbanicity and the DBM (Jones et al., 2016). Furthermore, a recent multilevel analysis on global inequalities in household‐level DBM (i.e., maternal overweight/obesity and child stunting) conducted across 55 LMICs (1992–2018) showed that the prevalence of household‐level DBM was higher among wealthier households in lower‐income countries (Seferidi et al., 2022).

The fact that undernutrition and overweight/obesity are biologically interlinked contributes to the coexistence of multiple forms of malnutrition at the individual or household levels, either at one point in time or across the life course. Some of the pathways through which different forms of malnutrition coexist have been described in detail elsewhere (Wells et al., 2020). Overweight/obesity during pregnancy may be associated with higher birthweight, but it may also result in child undernutrition (Barquera et al., 2007; Jones et al., 2016; Phillips et al., 2014; Wawer et al., 2021; Wells et al., 2020). For example, a recent study found that, in comparison to normal‐weight pregnant women, those with overweight/obesity failed to upregulate iron absorption in late pregnancy, and transferred less iron to their foetus, which led to their infants having lower body iron stores (Stoffel et al., 2022). Likewise, undernutrition during pregnancy can result in low birthweight (Figueiredo et al., 2018, 2019; Rahman et al., 2016; Wells et al., 2020). Exposure to undernutrition in early life may increase the risk of developing overweight/obesity and NCDs later in life, particularly in the context of obesogenic environments in countries undergoing the nutrition transition (Arage et al., 2021; Hoffman et al., 2000; Leon et al., 1996; Li et al., 2015; Victora et al., 2008; Wells et al., 2020).

Undernutrition and overweight/obesity share common biological drivers, and also social and environmental ones (Pradeilles et al., 2019). This underlines the need for countries like Ethiopia to adopt double‐duty actions that can address both forms of malnutrition simultaneously. Although the national prevalence of household‐level DBM is still relatively low, the prevalence and trend of household‐level DBM in urban areas such as Addis Ababa is alarming and illustrate the need to have bolder policies and programmes towards the prevention of overweight/obesity, while continuing to address undernutrition. The recently adopted Ethiopian Food and Nutrition Policy has highlighted the rising burden of overweight and obesity (Federal Democratic Republic of Ethiopia, 2018). Also, more recently, the Ethiopian Excise tax proclamation No. 1186/2020 has proposed an excise tax (up to 60%) on foods and beverages high in saturated/trans fats, sugar, and sodium (Federal Democratic Republic of Ethiopia, 2020). Although these measures are encouraging, interventions are still siloed by the forms of malnutrition they target, either undernutrition or overweight/obesity. For example, fiscal policies that subsidise sugar and oil have been put in place to support the poorest segment of the population, whereas at the national level, tax is levied on sugar. This is despite the fact that sugars can displace more nutrient‐dense foods and thus contribute to undernutrition as well (WHO, 2003). Besides, subsidies on healthier alternatives like fruits and vegetables do not exist yet. Regulations on the advertisement of unhealthy foods on billboards, TV, radio and so on are needed. In urban areas, food labelling (e.g., nutri‐score) can help residents make informed choices. Linking these interventions and prioritising them by their potential to address both forms of malnutrition would likely be more effective.

To our knowledge, this is the first study to provide an in‐depth and comprehensive situation analysis of the household‐level DBM in Ethiopia using three rounds of nationally representative surveys. The present study also comes with a number of limitations. First, although we have analysed the temporal trends in DBM using various DHS rounds, the study remains cross‐sectional. We used internationally agreed BMI cut‐offs to define overweight/obesity; however, it has been argued that the cut‐offs for obesity and markers of metabolic syndrome in Ethiopian adults are lower than the international values (Sinaga et al., 2018). Consequently, our DBM estimates could be a conservative estimate. Relatedly, we did not assess the combination of child overweight/obesity with maternal undernutrition as the prevalence of this combination was close to zero. This is supported by recent findings from Popkin et al. (2020) and Irache et al. (2022), who observed that the coexistence of child overweight/obesity and maternal undernutrition at the household level is very low in LMICs. Additionally, due to the availability of data only for WRA and children U5 in EDHS, we based our estimate of household‐level DBM using data only for mothers and children U5 living within the same household. WRA and children U5 experience the highest burden of malnutrition and hence we do not anticipate that the prevalence of household‐level DBM would change substantially if other household members were included in the derivation of DBM. Relevant dietary data to the DBM are not collected in the DHS meaning we were unable to assess the likely important contribution of changing diets (such as the consumption of energy‐dense and nutrient‐poor foods) to the change in the prevalence of DBM. Occupation is broadly categorised as income‐generating or not, with the former being further disaggregated into manual and nonmanual. The effect of manual and nonmanual work on the form of malnutrition may be different in urban and rural settings. Finally, we have examined trends in DBM up to 2016, which is the latest DHS for which the data is complete to estimate DBM. As such, we have been unable to capture any recent changes in the prevalence of DBM, which we expect to have increased further. While the mini 2019 EDHS report has been published, this does not provide all the necessary data required to generate estimates of DBM. A recent regional study in Ethiopia conducted in 2018 estimated the prevalence of household‐level DBM (defined as any adult overweight and child stunting) to be at 9.3% in Addis Ababa. The comparable 2016 prevalence estimate for Addis Ababa in our study was 2.8% and this disparity may be related to differences in the sampling procedure and in the criteria used to define DBM (Bliznashka et al., 2021).

## 5 CONCLUSION

The present study has highlighted that a relatively low prevalence of household‐level DBM at the national level can mask serious disparities among sub‐populations. The prevalence of household‐level DBM is significantly higher in urban areas and in households with higher wealth, calling for double‐duty actions that address both forms of malnutrition, particularly in urban areas. The prevalence of household‐level DBM is likely to increase and shift to poorer households as Ethiopia develops economically. Public health and nutrition interventions that address all forms of malnutrition and reduce disparities should be identified and prioritised. For example, improving diets by transforming food systems to make healthy diets available, accessible and affordable is critical. In addition, regulating the promotion and distribution of unhealthy foods, while promoting nutrient‐dense foods, should be prioritised and occur alongside behavioural change communications that instil healthier dietary behaviours.

## AUTHOR CONTRIBUTIONS

Rebecca Pradeilles and Kaleab Baye designed the scope of the paper. Rebecca Pradeilles, Ana Irache and Tom Norris conducted the analysis. Rebecca Pradeilles, Ana Irache, Tom Norris and Kaleab Baye wrote the first draft of the paper. Arnaud Laillou and Stanley Chitekwe reviewed the manuscript. All authors read and approved the final manuscript.

## CONFLICTS OF INTEREST

The authors declare no conflicts of interest.

## Supporting information

Supporting information.

## Data Availability

We used data from the Demographic and Health Surveys (DHS), which are publicly available and can be accessed from: https://dhsprogram.com.
