# Supplementary material for: Magnitude, trends and drivers of the coexistence of maternal overweight/obesity and childhood undernutrition in Ethiopia: Evidence from Demographic and Health Surveys (2005–2016)
Source: Matern Child Nutr. 2022 May 25;20(Suppl 5):e13372. doi: 10.1111/mcn.13372 (PMC11258774; doi:10.1111/mcn.13372)
Supplement: Supplementary file 1 — Supporting information. [file MCN-20-e13372-s001.docx]

**Supplemental Table 1** National trends in the different forms of malnutrition at the national level and by regions, and annualised change in prevalence between the latest and earliest survey years

|  | **2005 EDHS** | **2011 EDHS** | **2016 EDHS** | **Annualised change** |
| --- | --- | --- | --- | --- |
| **Overweight & obesity among women of reproductive age (15-49 years old)** | | | | |
| Tigray | 1.5 [0.8, 2.3] | 3.4 [2.0, 5.3] | 5.9 [4.3, 8.1] | 0.40 |
| Afar | 4.4 [2.0, 9.5] | 4.5 [3.1, 6.6] | 8.4 [6.0, 11.7] | 0.36 |
| Amhara | 2.5 [1.6, 4.0] | 3.7 [2.7, 5.2] | 3.6 [2.7, 4.8] | 0.10 |
| Oromia | 5.0 [3.3, 7.5] | 5.1 [3.8, 6.9] | 7.9 [5.6, 11.2] | 0.26 |
| Somali | 10.1 [5.3, 18.3] | 16.0 [11.3, 22.1] | 15.3 [12.0, 19.3] | 0.47 |
| Benishangul | 2.0 [0.9, 4.2] | 3.6 [2.1, 6.2] | 7.8 [5.8, 10.1] | 0.53 |
| SNNPR | 3.3 [2.4, 4.6] | 6.4 [4.7, 8.7] | 6.3 [4.7, 8.4] | 0.27 |
| Gambela | 2.7 [1.2, 6.2] | 7.8 [5.6, 10.8] | 8.7 [6.4, 11.9] | 0.55 |
| Harari | 10.3 [7.5, 13.9] | 15.3 [13.3, 17.4] | 20.6 [17.5, 24.2] | 0.93 |
| Addis Ababa | 18.2 [14.9, 21.9] | 20.2 [18.1, 22.5] | 30.5 [28.1, 32.9] | 1.12 |
| Dire Dawa | 14.7 [11.5, 18.6] | 18.7 [15.8, 22.4] | 22.4 [19.7, 25.3] | 0.70 |
| **Ethiopia** | **4.7 [3.9, 5.6]** | **6.0 [5.3, 6.8]** | **8.1 [7.0, 9.2]** | **0.31** |
| **Childhood stunting (0-59 months)** | | | | |
| Tigray | 47.8 [42.5, 53.2] | 50.1 [46.2, 54.1] | 39.1 [35.6, 42.8] | -0.79 |
| Afar | 39.2 [29.8, 49.4] | 46.9 [42.6, 51.3] | 39.9 [35.7, 44.3] | 0.06 |
| Amhara | 62.4 [56.6, 68.0] | 48.8 [45.5, 52.1] | 46.8 [42.8, 50.9] | -1.42 |
| Oromia | 43.7 [39.2, 48.4] | 40.5 [37.3, 43.7] | 36.0 [32.9, 39.2] | -0.70 |
| Somali | 48.2 [40.4, 56.0] | 31.7 [26.9, 37.0] | 26.7 [23.1, 30.5] | -1.95 |
| Benishangul | 43.0 [34.4, 52.1] | 47.0 [42.6, 51.4] | 40.7 [36.3, 45.3] | -0.21 |
| SNNPR | 52.1 [48.7, 55.4] | 42.0 [38.3, 45.9] | 38.4 [34.5, 42.3] | -1.25 |
| Gambela | 37.8 [28.9, 47.7] | 27.3 [23.0, 32.1] | 23.0 [19.1, 27.5] | -1.35 |
| Harari | 45.3 [38.3, 52.6] | 28.1 [23.3, 33.3] | 31.6 [26.8, 36.9] | -1.25 |
| Addis Ababa | 24.8 [17.1, 34.5] | 21.3 [16.8, 26.4] | 14.7 [11.1, 19.2] | -0.92 |
| Dire Dawa | 33.3 [25.9, 41.5] | 33.7 [28.5, 39.3] | 40.9 [34.5, 47.7] | 0.69 |
| **Ethiopia** | **49.7 [47.3, 52.0]** | **43.3 [41.5, 45.0]** | **38.0 [36.2, 39.9]** | **-1.06** |
| **Childhood anaemia (6-59 months)** | | | | |
| Tigray | 56.4 [50.4, 62.2] | 37.3 [33.6, 41.0] | 54.2 [49.3, 59.1] | -0.20 |
| Afar | 57.2 [40.4, 63.7] | 73.7 [69.3, 77.6] | 74.9 [70.0, 79.3] | 1.61 |
| Amhara | 49.8 [44.2, 55.5] | 34.1 [30.8, 37.6] | 42.7 [38.1, 47.3] | -0.65 |
| Oromia | 54.4 [50.0, 58.7] | 51.2 [47.0, 55.4] | 65.3 [60.8, 69.6] | 0.99 |
| Somali | 83.9 [75.0, 90.1] | 69.2 [64.3, 73.7] | 82.9 [79.4, 86.0] | -0.09 |
| Benishangul | 51.3 [50.8, 61.6] | 45.7 [40.9, 50.7] | 42.2 [36.8, 47.8] | -0.83 |
| SNNPR | 44.9 [40.2, 49.6] | 36.6 [33.3, 40.1] | 49.6 [44.4, 54.8] | 0.43 |
| Gambela | 61.0 [48.6, 72.1] | 50.5 [40.5, 60.4] | 56.5 [47.7, 64.9] | -0.41 |
| Harari | 58.3 [46.4, 69.2] | 54.3 [49.0, 59.5] | 66.1 [61.0, 70.9] | 0.71 |
| Addis Ababa | 40.6 [32.7, 49.1] | 32.9 [27.9, 38.4] | 48.4 [42.0, 54.9] | 0.71 |
| Dire Dawa | 60.7 [48.1, 72.0] | 62.2 [56.7, 67.4] | 71.1 [64.6, 76.8] | 0.95 |
| **Ethiopia** | **52.2 [49.7, 54.6]** | **43.7 [41.6, 45.8]** | **57.0 [54.5, 59.5]** | **0.44** |

*Annualised change= (prevalence 2016 – prevalence 2005) ÷ (2016 - 2005)

**Supplemental Table 1** National trends in the different forms of malnutrition at the national level and by regions, and annualised change in prevalence between the latest and earliest survey years (continued)

|  | **2005 EDHS** | **2011 EDHS** | **2016 EDHS** | **Annualised change** |
| --- | --- | --- | --- | --- |
| **Childhood wasting (0-59 months)** | | | | |
| Tigray | 13.6 [10.4, 17.6] | 10.9 [9.0, 13.1] | 11.6 [9.6, 13.8] | -0.18 |
| Afar | 15.1 [9.1, 24.1] | 21.4 [19.0, 24.1] | 18.7 [15.1, 22.9] | 0.33 |
| Amhara | 18.5 [15.4, 22.1] | 11.7 [9.6, 14.2] | 10.2 [8.3, 12.5] | -0.75 |
| Oromia | 12.0 [9.5, 15.0] | 10.4 [8.7, 12.4] | 10.8 [9.2, 12.7] | -0.11 |
| Somali | 23.8 [18.8, 29.6] | 22.7 [18.4, 27.6] | 23.8 [19.0, 29.3] | 0 |
| Benishangul | 22.8 [15.9, 31.6] | 10.7 [8.9, 12.9] | 10.9 [8.1, 14.4] | -1.08 |
| SNNPR | 9.6 [7.4, 12.4] | 8.5 [6.9, 10.4] | 6.6 [5.1, 8.5] | -0.27 |
| Gambela | 10.5 [6.2, 17.3] | 13.5 [9.8, 18.5] | 14.5 [10.9, 19.0] | 0.36 |
| Harari | 11.8 [7.0, 19.1] | 9.5 [6.8, 13.1] | 11.6 [8.3, 16.1] | -0.02 |
| Addis Ababa | 5.1 [2.3, 11.1] | 5.9 [3.3, 10.3] | 3.6 [2.2, 5.8] | -0.14 |
| Dire Dawa | 15.8 [11.7, 21.0] | 13.5 [10.6, 17.1] | 11.3 [8.3, 15.1] | -0.41 |
| **Ethiopia** | **13.5 [12.1, 15.0]** | **10.7 [9.7, 11.8]** | **10.4 [9.4, 11.4]** | **-0.28** |

*Annualised change= (prevalence 2016 – prevalence 2005) ÷ (2016 - 2005)

**Supplemental Table 2** Prevalence of intra-household DBM at the national level and by regions, and annualised change in prevalence between the latest and earliest survey years

|  | **2005 EDHS** | **2011 EDHS** | **2016 EDHS** | **Annualised change*** |
| --- | --- | --- | --- | --- |
| **Maternal overweight/obesity and U5 stunting** | | | | |
| Tigray | 1.0 [0.2, 4.5] | 0.8 [0.3, 2.6] | 1.2 [0.6, 2.7] | 0.02 |
| Afar | 1.6 [1.1, 2.3] | 1.6 [0.7, 3.8] | 3.5 [1.9, 6.3] | 0.17 |
| Amhara | 0.4 [0.1, 2.8] | 1.0 [0.5, 2.1] | 0.7 [0.2, 1.7] | 0.03 |
| Oromia | 2.6 [1.4, 5.1] | 1.0 [0.5, 2.1] | 1.2 [0.7, 2.3] | -0.13 |
| Somali | 4.5 [2.0, 9.8] | 6.6 [3.9, 11.2] | 3.7 [2.2, 6.2] | -0.07 |
| Benishangul | 0 | 0.6 [0.1, 2.5] | 1.5 [0.6, 3.8] | 0.14 |
| SNNPR | 1.1 [0.4, 3.0] | 1.0 [0.4, 2.6] | 1.7 [0.9, 3.2] | 0.05 |
| Gambela | 0.6 [0.5, 0.7] | 0.9 [0.2, 4.0] | 0.8 [0.3, 2.3] | 0.02 |
| Harari | 3.1 [1.0, 9.8] | 1.8 [0.8, 4.3] | 4.5 [2.4, 8.4] | 0.13 |
| Addis Ababa | 6.0 [2.4, 14.1] | 4.5 [2.6, 7.6] | 3.8 [2.2, 6.6] | -0.20 |
| Dire Dawa | 3.4 [1.0, 10.8] | 4.0 [2.0, 7.8] | 5.4 [3.4, 8.7] | 0.18 |
| **Ethiopia** | **1.7 [1.1, 2.6]** | **1.4 [1.0, 2.0]** | **1.5 [1.1, 2.1]** | **-0.02** |
| **Maternal overweight/obesity and U5 anaemia** | | | | |
| Tigray | 0.4 [0.1, 3.2] | 1.2 [0.4, 3.2] | 1.7 [0.8, 3.4] | 0.12 |
| Afar | 0 | 1.9 [0.8, 4.2] | 3.6 [2.0, 6.2] | 0.33 |
| Amhara | 0.8 [0.2, 3.1] | 0.3 [0.1, 1.1] | 1.5 [0.6, 3.6] | 0.06 |
| Oromia | 1.5 [0.5, 4.0] | 1.5 [0.8, 2.8] | 2.6 [1.6, 4.1] | 0.10 |
| Somali | 5.9 [2.6, 12.8] | 10.2 [5.8, 17.3] | 11.6 [7.6, 17.2] | 0.52 |
| Benishangul | 0 | 0.2 [0.0, 1.5] | 1.9 [0.9, 4.1] | 0.17 |
| SNNPR | 1.9 [1.1, 3.4] | 1.4 [0.6, 3.0] | 2.8 [1.7, 4.5] | 0.08 |
| Gambela | 3.2 [0.9, 10.5] | 1.4 [0.4, 4.7] | 2.7 [1.1, 6.8] | -0.05 |
| Harari | 5.3 [1.9, 14.2] | 6.7 [4.2, 10.7] | 8.9 [5.3, 14.5] | 0.33 |
| Addis Ababa | 7.5 [3.0, 17.8] | 7.9 [4.9, 12.6] | 24.9 [18.9, 32.0] | 1.58 |
| Dire Dawa | 5.7 [2.2, 14.3] | 7.5 [4.5, 12.1] | 9.8 [6.0, 15.8] | 0.37 |
| **Ethiopia** | **1.6 [1.0, 2.4]** | **1.4 [1.0, 2.0]** | **3.1 [2.5, 3.9]** | **0.14** |
| **Maternal overweight/obesity and U5 wasting** | | | | |
| Tigray | 0 | 0.2 [0.0, 1.5] | 0 | 0 |
| Afar | 0 | 0.3 [0.0, 2.6] | 0.9 [0.3, 2.9] | 0.08 |
| Amhara | 0.6 [0.2, 2.6] | 0.4 [0.1, 1.5] | 0 | -0.05 |
| Oromia | 0 | 0.2 [0.1, 0.9] | 0.2 [0.1, 1.0] | 0.02 |
| Somali | 0.5 [0.1, 4.2] | 2.2 [1.0, 4.7] | 3.0 [1.8, 5.1] | 0.23 |
| Benishangul | 0 | 0 | 0.3 [0.0, 2.0] | 0.03 |
| SNNPR | 0 | 0.5 [0.2, 1.5] | 0 | 0 |
| Gambela | 0.5 [0.4, 0.7] | 0 | 1.7 [0.5, 5.9] | 0.11 |
| Harari | 0 | 0.4 [0.1, 2.8] | 0.4 [0.0, 2.8] | 0.04 |
| Addis Ababa | 0 | 0.3 [0.0, 2.6] | 1.2 [0.4, 3.2] | 0.11 |
| Dire Dawa | 0 | 1.0 [0.3, 3.3] | 0.3 [0.0, 2.4] | 0.03 |
| **Ethiopia** | **0.2 [0.1, 0.6]** | **0.4 [0.2, 0.7]** | **0.2 [0.1, 0.4]** | **0** |

*Annualised change= (DBM prevalence 2016 – DBM prevalence 2005) ÷ (2016 - 2005)

**Supplemental Table 2** Prevalence of intra-household DBM at the national level and by regions, and annualised change in prevalence between the latest and earliest survey years (continued)

|  | **2005 EDHS** | **2011 EDHS** | **2016 EDHS** | **Annualised change*** |
| --- | --- | --- | --- | --- |
| **Any form of DBM (Maternal overweight/obesity and U5 anaemia or stunting)** | | | | |
| Tigray | 0.9 [0.2, 4.2] | 1.9 [0.9, 3.7] | 2.9 [1.8, 4.6] | 0.18 |
| Afar | 1.3 [0.9, 1.9] | 2.4 [1.3, 4.5] | 4.8 [2.7, 8.3] | 0.32 |
| Amhara | 1.0 [0.3, 3.1] | 1.2 [0.6, 2.3] | 1.7 [0.8, 3.7] | 0.06 |
| Oromia | 3.3 [1.8, 5.9] | 1.6 [0.9, 2.7] | 2.9 [1.9, 4.3] | -0.04 |
| Somali | 4.7 [2.2, 9.8] | 11.4 [6.9, 18.2] | 11.8 [8.5, 16.2] | 0.65 |
| Benishangul | 0 | 0.5 [0.1, 2.4] | 2.7 [1.2, 6.1] | 0.25 |
| SNNPR | 2.1 [1.2, 3.5] | 2.2 [1.2, 4.0] | 3.3 [2.0, 5.3] | 0.11 |
| Gambela | 2.5 [0.7, 8.5] | 1.5 [0.5, 4.3] | 2.7 [1.1, 6.3] | 0.02 |
| Harari | 5.4 [2.2, 12.5] | 6.9 [4.5, 10.2] | 9.9 [6.3, 15.1] | 0.41 |
| Addis Ababa | 8.9 [4.3, 17.2] | 8.6 [5.8, 12.6] | 22.8 [17.6, 28.9] | 1.26 |
| Dire Dawa | 5.3 [2.3, 11.9] | 8.3 [5.3, 12.8] | 9.7 [6.4, 14.5] | 0.40 |
| **Ethiopia** | **2.4 [1.7, 3.4]** | **2.0 [1.6, 2.6]** | **3.5 [2.9, 4.3]** | **0.10** |

*Annualised change= (DBM prevalence 2016 – DBM prevalence 2005) ÷ (2016 - 2005)

**Supplemental Table 3** Description of variables included in the construction of the SWPER index

| **DHS questions** | **Code** |
| --- | --- |
| **Domain 1: Attitude towards Violence** | |
| Beating justified if wife goes out without telling husband | Justified= -1; don’t know=0; not justified =1 |
| Beating justified if wife neglects the children | Justified= -1; don’t know=0; not justified =1 |
| Beating justified if wife argues with husband | Justified= -1; don’t know=0; not justified =1 |
| Beating justified if wife refuses to have sex with husband | Justified= -1; don’t know=0; not justified =1 |
| Beating justified if wife burns the food | Justified= -1; don’t know=0; not justified =1 |
| **Domain 2: Social independence** | |
| Frequency of reading newspaper or magazine | Not at all=0; <once a week=1; ≥once a week=2 |
| Respondent worked in last 12 months | No=0; in the past year=1; have a job, but on leave past 7 days=2; currently working=2 |
| Woman’s education | Years |
| Education difference: woman’s minus husband’s years of Schooling | Years |
| Age difference: woman’s minus husband’s age | Years |
| Age at first cohabitation | Years |
| Age of respondent at 1st birth | Years |
| **Domain 3: Decision making** | |
| Who usually decides on respondent's health care | Husband or other alone= –1; joint=0; respondent alone=1 |
| Who usually decides on large household purchases | Husband or other alone= –1; joint=0; respondent alone=1 |
| Who usually decides on visits to family or relatives | Husband or other alone= –1; joint=0; respondent alone=1 |

**Supplemental Table 4** Inequalities in the distribution of household level DBM by household wealth

|  | **2005 EDHS** | **2011 EDHS** | **2016 EDHS** |
| --- | --- | --- | --- |
| **Maternal overweight/obesity and U5 stunting** | | | |
| T1: Poorest/Poorer | 1.6 [0.9, 2.8] | 0.9 [0.5, 1.6] | 1.2 [0.7, 2.0] |
| T2: Middle | 0.6 [0.1, 4.0] | 0.6 [0.2, 1.6] | 0.4 [0.1, 1.4] |
| T3: Richer/Richest | 2.4 [1.5, 4.0] | 2.0 [1.3, 3.0] | 2.2 [1.5, 3.2] |
| **Inequality gap*** | -0.8 | -1.1 | -1.0 |
| **Maternal overweight/obesity and U5 anaemia** | | | |
| T1: Poorest/Poorer | 1.7 [1.0, 2.6] | 0.8 [0.4, 1.5] | 1.3 [0.8, 2.1] |
| T2: Middle | 0.6 [0.5, 0.6] | 0.3 [0.1, 1.2] | 1.7 [0.9, 3.2] |
| T3: Richer/Richest | 2.0 [1.1, 3.9] | 2.8 [2.0, 4.0] | 6.1 [4.7, 7.8] |
| **Inequality gap** | -0.3 | -2.0 | -4.8 |
| **Any form of DBM (Maternal overweight/obesity and U5 anaemia or stunting)** | | | |
| T1: Poorest/Poorer | 1.9 [1.2, 3.2] | 1.0 [0.6, 1.8] | 1.8 [1.2, 2.7] |
| T2: Middle | 1.3 [0.5, 3.1] | 0.9 [0.4, 2.1] | 1.7 [0.9, 3.1] |
| T3: Richer/Richest | 3.6 [2.4, 5.4] | 3.8 [2.8, 5.1] | 6.6 [5.3, 8.3] |
| **Inequality gap** | -1.7 | -2.8 | -4.8 |

*Inequality gap= (DBM Prevalence T1– DBM Prevalence T3)

**Supplemental Table 5** Inequalities in the distribution of household level DBM by maternal education level

|  | **2005 EDHS** | **2011 EDHS** | **2016 EDHS** |
| --- | --- | --- | --- |
| **Maternal overweight/obesity and U5 stunting** | | | |
| E1: No education | 1.6 [1.0, 2.8] | 1.1 [0.7, 1.6] | 1.5 [1.0, 2.3] |
| E2: Primary | 1.0 [0.3, 3.2] | 1.5 [0.8, 2.8] | 1.1 [0.6, 1.9] |
| E3: Secondary+ | 5.0 [2.3, 10.9] | 1.2 [0.6, 2.6] | 1.3 [0.6, 2.9] |
| **Inequality gap*** | -3.4 | -0.1 | 0.2 |
| **Maternal overweight/obesity and U5 anaemia** | | | |
| E1: No education | 1.2 [0.7, 2.0] | 1.1 [0.7, 1.7] | 1.9 [1.4, 2.6] |
| E2: Primary | 1.0 [0.4, 2.2] | 1.6 [1.0, 2.5] | 4.0 [2.8, 5.8] |
| E3: Secondary+ | 10.3 [4.0, 23.9] | 5.5 [2.7, 11.2] | 10.0 [6.8, 14.3] |
| **Inequality gap** | -9.1 | -4.4 | -8.1 |
| **Any form of DBM (Maternal overweight/obesity and U5 anaemia or stunting)** | | | |
| E1: No education | 1.9 [1.2, 3.0] | 1.6 [1.1, 2.2] | 2.4 [1.8, 3.2] |
| E2: Primary | 2.1 [1.1, 3.8] | 2.6 [1.7, 4.0] | 4.3 [3.0, 6.0] |
| E3: Secondary+ | 10.2 [4.8, 20.5] | 5.1 [2.7, 9.4] | 9.6 [6.7, 13.5] |
| **Inequality gap** | -8.3 | -3.5 | -7.2 |

*Inequality gap= (DBM Prevalence E1– DBM Prevalence E3)

**Supplemental Table 6** Inequalities in the distribution of household level DBM by area of residence

|  | **2005 EDHS** | **2011 EDHS** | **2016 EDHS** |
| --- | --- | --- | --- |
| **Maternal overweight/obesity and U5 stunting** | | | |
| Addis Ababa | 6.2 [2.5, 14.5] | 4.5 [2.6, 7.6] | 3.8 [2.2, 6.6] |
| Dire Dawa | 4.9 [1.0, 19.9] | 6.5 [3.0, 13.8] | 9.5 [5.9, 14.8] |
| Other urban | 5.4 [2.5, 11.6] | 3.2 [1.7, 5.9] | 3.1 [1.8, 5.4] |
| Urban (all) | 5.6 [3.0, 10.1] | 3.4 [2.1, 5.6] | 3.3 [2.2, 5.1] |
| Rural | 1.3 [0.7, 2.3] | 0.8 [0.5, 1.3] | 1.1 [0.7, 1.6] |
| **Inequality gap*** | -4.3 | -2.6 | -2.2 |
| **Maternal overweight/obesity and U5 anaemia** | | | |
| Addis Ababa | 7.9 [3.1, 18.8] | 7.9 [4.9, 12.6] | 24.9 [18.9, 32.0] |
| Dire Dawa | 11.8 [4.4, 28.1] | 12.0 [6.7, 20.5] | 18.1 [10.3, 29.9] |
| Other urban | 5.5 [1.5, 17.9] | 3.7 [2.2, 6.4] | 9.5 [6.2, 14.2] |
| Urban (all) | 6.0 [2.4, 14.7] | 4.4 [2.9, 6.6] | 12.5 [9.4, 16.4] |
| Rural | 1.2 [0.7, 1.9] | 0.9 [0.6, 1.5] | 1.7 [1.2, 2.4] |
| **Inequality gap** | -4.8 | -3.5 | -10.8 |
| **Any form of DBM (Maternal overweight/obesity and U5 anaemia or stunting)** | | | |
| Addis Ababa | 9.1 [4.5, 17.7] | 8.6 [5.8, 12.6] | 22.8 [17.6, 28.9] |
| Dire Dawa | 8.7 [3.3, 21.1] | 13.5 [8.1, 21.6] | 16.7 [10.6, 25.3] |
| Other urban | 8.2 [3.7, 17.2] | 5.5 [3.5, 8.6] | 10.3 [7.1, 14.8] |
| Urban (all) | 8.4 [4.5, 14.9] | 6.1 [4.3, 8.6] | 12.9 [10.0, 16.5] |
| Rural | 1.8 [1.2, 2.7] | 1.3 [0.9, 1.9] | 2.1 [1.6, 2.8] |
| **Inequality gap** | -6.6 | -4.8 | -10.8 |

*Inequality gap= (DBM Prevalence rural – DBM Prevalence urban all)

**Supplemental Table 7** Factors associated with individual forms of malnutrition: pooled univariable regression models of EDHS 2005, 2011 and 2016

| **Factors** | |  | **Child stunting (n=11,079)** | | |  | **Child anaemia (n=11,033)** | | |  | **Maternal overweight/obesity**  **(n=11,080)** | | |
| --- | --- | --- | --- | --- | --- | --- | --- | --- | --- | --- | --- | --- | --- |
|  |  |  | **OR** | **95% CI** | **P-value** |  | **OR** | **95% CI** | **P-value** |  | **OR** | **95% CI** | **P-value** |
| Maternal age (years) | |  | 1.00 | 1.00, 1.01 | 0.001 |  | 0.98 | 0.98, 0.99 | <0.001 |  | 1.04 | 1.03, 1.05 | <0.001 |
| Residence: Urban | |  | 0.45 | 0.41, 0.50 | <0.001 |  | 0.72 | 0.66, 0.79 | <0.001 |  | 10.21 | 8.73, 11.9 | <0.001 |
| Maternal literacy | |  |  |  |  |  |  |  |  |  |  |  |  |
| *Cannot read at all* | |  | Ref | _ | _ |  | Ref | _ | _ |  | Ref | _ | _ |
| *Able to read only parts of sentence* | |  | 0.75 | 0.66, 0.87 | <0.001 |  | 0.80 | 0.70, 0.91 | 0.001 |  | 2.17 | 1.69, 2.78 | <0.001 |
| *Able to read whole sentence* | |  | 0.46 | 0.41, 0.52 | <0.001 |  | 0.66 | 0.59, 0.73 | <0.001 |  | 5.72 | 4.87, 6.71 | <0.001 |
| Maternal Occupation | |  |  |  |  |  |  |  |  |  |  |  |  |
| *Not working* | |  | Ref | _ | _ |  | Ref | _ | _ |  | Ref | _ | _ |
| *Non-manual* | |  | 0.79 | 0.71, 0.88 | <0.001 |  | 0.6 | 0.54, 0.66 | <0.001 |  | 1.98 | 1.68, 2.34 | <0.001 |
| *Manual* |  |  | 1.28 | 1.18, 1.40 | <0.001 |  | 0.63 | 0.58, 0.69 | <0.001 |  | 0.37 | 0.29, 0.47 | <0.001 |
| Paternal Occupation | |  |  |  |  |  |  |  |  |  |  |  |  |
| *Not working* | |  | Ref | _ | _ |  | Ref | _ | _ |  | Ref | _ | _ |
| *Non-manual* | |  | 0.85 | 0.69, 1.05 | 0.125 |  | 0.68 | 0.56, 0.83 | <0.001 |  | 1.84 | 1.35, 2.51 | <0.001 |
| *Manual* | |  | 1.51 | 1.25, 1.82 | <0.001 |  | 0.72 | 0.60, 0.87 | 0.001 |  | 0.37 | 0.27, 0.50 | <0.001 |
| Wealth score (0-10) | |  | 0.79 | 0.77, 0.81 | <0.001 |  | 0.88 | 0.86, 0.90 | <0.001 |  | 1.63 | 1.58, 1.69 | <0.001 |
| Number of children < 5 in household | |  | 0.99 | 0.94, 1.05 | 0.839 |  | 1.33 | 1.26, 1.40 | <0.001 |  | 0.83 | 0.75, 0.92 | 0.001 |
| Number of WRA in household | |  | 0.91 | 0.85, 0.97 | 0.004 |  | 0.86 | 0.81, 0.91 | <0.001 |  | 1.66 | 1.52, 1.82 | <0.001 |
| Sex of household head: Female | |  | 0.95 | 0.87, 1.05 | 0.331 |  | 1.11 | 1.01, 1.22 | 0.028 |  | 1.46 | 1.24, 1.73 | <0.001 |
| Age of household head (years) | |  | 1.00 | 1.00, 1.01 | 0.004 |  | 0.99 | 0.99, 0.99 | <0.001 |  | 1.01 | 1.00, 1.02 | <0.001 |
| Maternal education (years) | |  | 0.90 | 0.89, 0.91 | <0.001 |  | 0.96 | 0.95, 0.97 | <0.001 |  | 1.20 | 1.18, 1.21 | <0.001 |
| Paternal education (years) | |  | 0.91 | 0.91, 0.92 | <0.001 |  | 0.98 | 0.97, 0.99 | <0.001 |  | 1.16 | 1.15, 1.18 | <0.001 |
| SWPER: Autonomy/Social independence | |  | 0.77 | 0.73, 0.81 | <0.001 |  | 0.97 | 0.93, 1.02 | 0.294 |  | 2.09 | 1.93, 2.26 | <0.001 |
| SWPER: Decision making | |  | 0.85 | 0.81, 0.89 | <0.001 |  | 0.94 | 0.89, 0.98 | 0.005 |  | 1.79 | 1.63, 1.96 | <0.001 |

SWPER: survey-based women’s empowerment index; WRA: women of reproductive age

**Supplemental Table 8** Factors associated with individual forms of malnutrition: pooled multivariable regression models of EDHS 2005, 2011 and 2016

| **Factors** | | **Child stunting (n=9,357)** | | |  | **Child anaemia (n=9,319)** | | |  | **Maternal overweight & obesity**  **(n=9,358)** | | |
| --- | --- | --- | --- | --- | --- | --- | --- | --- | --- | --- | --- | --- |
|  |  | **OR** | **95% CI** | **P-value** |  | **OR** | **95% CI** | **P-value** |  | **OR** | **95% CI** | **P-value** |
| Maternal age (years) | | 0.99 | 0.99, 1.00 | 0.602 |  | 0.98 | 0.97, 0.99 | <0.001 |  | 1.06 | 1.04, 1.08 | <0.001 |
| Residence: Urban | | 1.06 | 0.88, 1.27 | 0.562 |  | 0.81 | 0.67, 0.97 | 0.023 |  | 3.20 | 2.39, 4.29 | <0.001 |
| Maternal literacy | |  |  |  |  |  |  |  |  |  |  |  |
| *Cannot read at all* | | Ref | _ | _ |  | Ref | _ | _ |  | Ref | _ | _ |
| *Able to read only parts of sentence* | | 1.10 | 0.93, 1.31 | 0.273 |  | 0.94 | 0.79, 1.11 | 0.509 |  | 1.41 | 1.01, 1.98 | 0.043 |
| *Able to read whole sentence* | | 0.95 | 0.76, 1.19 | 0.684 |  | 0.91 | 0.73, 1.13 | 0.405 |  | 1.58 | 1.06, 2.33 | 0.023 |
| Maternal Occupation | |  |  |  |  |  |  |  |  |  |  |  |
| *Not working* | | Ref | _ | _ |  | Ref | _ | _ |  | Ref | _ | _ |
| *Non-manual* | | 1.01 | 0.88, 1.15 | 0.778 |  | 0.72 | 0.64, 0.82 | <0.001 |  | 0.94 | 0.75, 1.18 | 0.613 |
| *Manual* | | 1.11 | 0.99, 1.23 | 0.054 |  | 0.84 | 0.75, 0.93 | 0.001 |  | 0.70 | 0.52, 0.93 | 0.016 |
| Paternal Occupation | |  |  |  |  |  |  |  |  |  |  |  |
| *Not working* | | Ref | _ | _ |  | Ref | _ | _ |  | Ref | _ | _ |
| *Non-manual* | | 1.06 | 0.84, 1.34 | 0.593 |  | 0.99 | 0.78, 1.25 | 0.913 |  | 0.89 | 0.61, 1.31 | 0.571 |
| *Manual* |  | 1.14 | 0.92, 1.39 | 0.231 |  | 1.02 | 0.82, 1.26 | 0.872 |  | 0.59 | 0.41, 0.85 | 0.005 |
| Wealth score (0-10) | | 0.85 | 0.81, 0.90 | <0.001 |  | 0.89 | 0.85, 0.94 | <0.001 |  | 1.24 | 1.15, 1.32 | <0.001 |
| Number of children < 5 in household | | 0.96 | 0.90, 1.02 | 0.174 |  | 1.14 | 1.07, 1.21 | <0.001 |  | 1.10 | 0.96, 1.26 | 0.153 |
| Number of WRA in household | | 0.96 | 0.88, 1.04 | 0.344 |  | 0.91 | 0.84, 0.99 | 0.030 |  | 1.22 | 1.07, 1.39 | 0.003 |
| Sex of household head: Female | | 1.08 | 0.95, 1.23 | 0.247 |  | 1.09 | 0.95, 1.25 | 0.197 |  | 1.04 | 0.82, 1.32 | 0.712 |
| Age of household head (years) | | 1.00 | 0.99, 1.00 | 0.057 |  | 0.99 | 0.99, 1.00 | 0.976 |  | 1.00 | 0.99, 1.01 | 0.273 |
| Maternal education (years) | | 0.97 | 0.95, 1.00 | 0.103 |  | 0.98 | 0.96, 1.01 | 0.308 |  | 1.01 | 0.97, 1.06 | 0.605 |
| Paternal education (years) | | 0.96 | 0.95, 0.98 | <0.001 |  | 1.00 | 0.99, 1.02 | 0.308 |  | 1.01 | 0.98, 1.04 | 0.366 |
| SWPER: Autonomy/Social independence | | 0.98 | 0.91, 1.06 | 0.912 |  | 1.11 | 1.03, 1.20 | 0.003 |  | 0.92 | 0.80, 1.04 | 0.203 |
| SWPER: Decision making | | 0.95 | 0.90, 1.00 | 0.090 |  | 1.06 | 1.00, 1.12 | 0.049 |  | 1.15 | 1.02, 1.29 | 0.021 |

Each variable in the multivariable models were mutually adjusted for all other variables including regions; SWPER: survey-based women’s empowerment index; WRA: women of reproductive age

**Supplemental Table 9** Contribution of the different factors to the change in child stunting and maternal overweight/obesity between 2005 and 2016

| **Drivers** | **Child stunting (n=5,285)** | | **Maternal overweight/obesity (n=5,285)** | |
| --- | --- | --- | --- | --- |
|  | **Contribution** | **P-value** | **Contribution** | **P-value** |
| Urban residence | 2.1% | 0.008 | 10.2% | <0.001 |
| Region | 4.8% | <0.001 | 13.7% | <0.001 |
| Maternal literacy | 0.1% | 0.858 | 3.8% | 0.067 |
| Maternal occupation | -1.4% | 0.035 | -0.5% | 0.067 |
| Wealth score (0-10) | 14.4% | <0.001 | 32.1% | <0.001 |
| Number of children <5 in household | -0.4% | 0.341 | -0.5% | 0.44 |
| Number of WRA in household | -0.3% | 0.273 | -1.2% | <0.001 |
| Sex of household head | -0.4% | 0.6 | 0.0% | 0.973 |
| Maternal education (single years) | 5.2% | 0.086 | -5.6% | 0.13 |
| SWPER: Autonomy/Social independence | 0.2% | 0.863 | -0.7% | 0.674 |
| SWPER: Decision making | 3.9% | 0.041 | 3.4% | 0.252 |
| Paternal occupation | 3.2% | 0.037 | 9.8% | 0.012 |
| Unexplained | 68.5% | <0.001 | 35.6% | 0.019 |

Note: Decomposition analyses could not be performed for child anaemia as the prevalence of anaemia between 2005 and 2016 did not substantially change; SWPER: survey-based women’s empowerment index; WRA: women of reproductive age

**Supplemental Figure 1** Participants’ flow diagram
